# Effect of silver diammine fluoride on micro-ecology of plaque from extensive caries of deciduous teeth - in vitro study

**DOI:** 10.1186/s12903-020-01141-3

**Published:** 2020-05-24

**Authors:** Bao ying Liu, Jin Liu, Di Zhang, Zhi lei Yang, Ya ping Feng, Meng Wang

**Affiliations:** 1 The First Affiliated Hospital of Zhengzhou University (School and Hospital of Stomatology), No 1 Jianshe Road, Pingdingshan, 450001 Henan Province China; 2 Academy of Medical Science, Zhengzhou University, Zhengzhou, Henan Province China; 3 The Second Affiliated Hospital of Pingdingshan College, Pingdingshan, Henan Province China

**Keywords:** Silver diammine fluoride, Plaque micro-ecology, Deciduous tooth, Dental caries, School children

## Abstract

**Background:**

The mechanism of action of silver diammine fluoride (SDF) on plaque micro-ecology is seldom studied. This study investigated micro-ecological changes in dental plaque on extensive caries of deciduous teeth after topical SDF treatment.

**Methods:**

Deciduous teeth with extensive caries freshly removed from school children were collected in clinic. Unstimulated saliva collection and initial plaque sampling were done before tooth extraction, then each caries was topically treated with 38% SDF in vitro. After intervention, each tooth was stored respectively in artificial saliva at 37 °C. Repeated plaque collections were done at 24 h and 1 week post-intervention. Post-intervention micro-ecological changes including microbial diversity, microbial metabolism function as well as species correlations were analyzed and compared after pyrosequencing of the DNA from the plaque sample using Illumina MiSeq platform.

**Results:**

After SDF application, microbial diversity decreased (*P* > 0.05), although not statistically significant. Microbial community composition post-intervention was noticeably different from that of supragingival and pre-intervention plaque as well as saliva. At 1 week post-intervention, the relative content of *Pseudomonas*, *Fusobacterium* and *Pseudoramibacter* were higher than before, while most of the other bacteria were reduced, although the changes were not statistically significant (*P* > 0.05). The inter-microbial associations became more complex, much more positive associations among survived bacteria were observed than negative ones. COG function classification diagram showed carbohydrate transportation and metabolic functions in the plaque were significantly reduced at 24 h and 1 week post-intervention.

**Conclusions:**

SDF has extensive antimicrobial effect on dental plaque, which may reduce carbohydrate metabolism in dental plaque and help promote new balance of the plaque flora.

## Background

Untreated deciduous caries affect 573 million children worldwide [1–3], jeopardize their health and increases the burden of medical care [4]. The incidence of permanent tooth decay increases with the severity of deciduous tooth decay [5] . Fluoride is an important effective weapon in fighting dental caries [6, 7]. Silver diammine fluoride (SDF) garnered great attention because its super ability in arresting and preventing caries [8–13]. It has been called the ‘silver bullet’ for dental caries [14].

Previous studies on the working mechanism of SDF in the control of dental caries focused mainly on a few specific cariogenic bacteria [15–20], whereas according to the concept of ‘ecological plaque hypothesis’ [21–24], caries development is due to the loss of micro-ecology balance within the dental plaque. New evidence suggests that SDF has no specific selectivity in inhibiting bacteria [25]. The effect of SDF on micro-ecology of dental plaque should be further investigated.

The aim of this study was to explore the effect of SDF on micro-ecology of carious dental biofilm.

## Methods

An in vitro study using clinic samples from school children was designed. Ethic approval was gain from the ethics committee of The First Affiliated Hospital of Zhengzhou University (NO. 2018-ky-35). Written informed consent from parents or guardians were obtained prior to any clinical examination or sampling.

### Subjects

Children aged 6–12 years admitted to Department of Pediatric Dentistry and Department of Oral and Maxillofacial Surgery in The Second Affiliated Hospital of Pingdingshan College in Henan province, during the period from November 2018 to January 2019 were targeted. Children were examined in the clinic and those with at least one deciduous tooth with extensive untreated caries were recruited.

Inclusion criteria: (1) children aged 6–12 years; (2) the presence of at least one deciduous tooth with extensive caries in the mouth, which needs to be extracted. Exclusion criteria: (1) the extensive caries was restored; (2) the surface of the caries was smooth and hard; (3) children with hereditary or systemic diseases; (4) children with a long history of medication or who have taken antibiotics within the last 3 months; (5) children who received topical fluoride therapy within the last 2 weeks.

### Collection of plaque and saliva before SDF intervention

Dental plaque on the tooth surface within the extensive untreated caries (PBI, Plaque Before Intervention), supragingival plaque on smooth surface of permanent first molars (PCF, Plaque from Caries-Free surface) and unstimulated saliva (S, Saliva) were collected from recruited children before tooth extraction. Sterilized dental excavators and sterilized scalers were used for collection of dental plaque within the caries and on sound surfaces, respectively. The collected plaque was put into a 1.5 ml centrifuge tube containing 1 ml PBS solution. Saliva in the amount of 2.5–3 ml was collected using the spitting method described by Fang Yang et al. [26]. All collected samples were immediately stored in a freezer at − 20 °C before laboratory analysis.

### SDF intervention

After extraction, the tooth was carefully cleaned on a sterile petri dish using sterile water and cotton to avoid blood contamination to the caries. An average amount of 10 μl SDF solution was topically applied onto dental surface within the extensive untreated caries by using a pipet, and avoiding touching the surface. A 38% (weight/volume percentage) SDF solution was used in this study, which was prepared in the laboratory of Zhengzhou University according to the method described previously [27]. Briefly, it contained NH_4_F 12.5 g, Ag_2_O 28 g, ammonia (26% NH_3_) 30 ml in 100 ml distilled water. This custom made SDF solution contains a concentration of 2.36 M (mole/l) silver fluoride in the solution, which is similar to a commonly used commercial product with 38% SDF (Saforide, Toyo Seiyaku Kasei, Osaka, Japan) [20]. The tooth was tilted around and assisted with a gentle blow to spread the solution as uniformly as possible on the carious surface. Each tooth was left undisturbed for 5 min in SDF before fully submerging into 10 ml of fresh, reduced sterile artificial saliva (Dongguan Yunfei Automation Equipment Technology Co., Ltd., Guangdong, China) in a sterile 50 ml tube and stored in a 37 °C incubator in ambient air. This artificial saliva contained NaCl 0.4 g, KCl 0.4 g, CaCl_2_·2H_2_O 0.795 g, NaH_2_PO_4_·H_2_O 0.69 g, Na_2_S·9H_2_O 0.005 g, KSCN 0.3 g, Urea 1 g in 1000 ml distilled water. Each tooth was transferred to fresh artificial saliva every 24 h and after plaque sampling procedures.

### Collection of plaque after SDF intervention

At the time of 24 h and 1 week after SDF intervention, dental plaque within the extensive untreated caries (PAI_24h, Plaque from caries 24 h After SDF Intervention; PAI_1w, Plaque from caries 1 week After SDF Intervention) was collected and stored again using the same method as mentioned above.

### Laboratory processing and analysis

Samples were sent out to Shanghai Majorbio Bio-pharm Technology Co. Ltd., China for laboratory procedures including DNA extraction, PCR amplification, 16S rDNA sequencing using the Illumina MiSeq platform and sequencing data processing. Laboratory processing was conducted according to standard operating procedures [28]. To ensure the quality of the samples, according to the PCR amplification results provided by this company, samples of quality grade ‘C’ were or would be discarded.

### Statistical analysis

Sequenced dilution curves, alpha diversity, and microbial species counts were calculated and analyzed on the free Majorbio online platform (www.majorbio.com). Bar plot and PCoA diagram were used where appropriate for other qualitative data. PCoA (principal co-ordinates analysis) was used to visually display the microbial diversity of different samples and to display the qualitative data in a quantitative manner. The values on the coordinate lines represent the relative degree of the difference from point to point. Student’s *t* test was used for pairwise comparison of sample alpha diversity between groups, and the Kruskal-Wallis rank sum test was used for comparison of sample species composition. Network analysis was used to analyze the relationship between bacteria genera. Network analysis uses various mathematic models such as similarity calculation, regression or Bayesian network analysis to build association network of a set of data. In microbial studies, analyzing high throughput sequencing data using mathematic similarity calculation based on Pearson or Spearman correlation coefficient is usually used to visual display the association network among different Operational Taxonomic Units (OTU) within the microbial sample. In the network diagram, each node represents one OTU, the size of the node represents the OTU abundance, the color of the node is set by the Majorbio online platform according to the advanced taxonomy level to which the OTU belongs. A line between two nodes implies a correlation, the red line is a positive correlation, the green line is a negative correlation, the more the lines, the closer the species is to other species. PICRUSt package (Phylogenetic Investigation of Communities by Reconstruction of Unobserved States) was used to analyze the functional characteristics of microorganisms in each group. PICRUSt is a bioinformatics software package freely available from http://picrust.github.io/picrust/, it can predict metagenome functional content from 16S rRNA gene sequencing data. Original version of PICRUSt was used in this study. The statistically significant level was set at *P* < 0.05.

## Results

In total, five children were recruited, general demographic and oral health status information of them are shown in Table 1. Each donated one extracted deciduous molar with an extensive untreated caries. A total of 15 plaque samples from carious lesions, 5 saliva samples and 5 supragingival plaque samples were collected. No sample presented a quality grade of ‘C’ as shown by PCR amplification results, therefore, all samples passed qualification test for further DNA sequencing.

**Table 1:** Age ranges and oral health status of the five children in this study

Subjects	Age ranges	Number of permanent / deciduous tooth	DMFT^a^	dmft^b^
NO.1	8 ~ 10	10 / 14	4	13
NO.2	8 ~ 10	12 / 10	3	10
NO.3	8 ~ 10	12 / 12	0	5
NO.4	8 ~ 10	14 / 10	0	6
NO.5	8 ~ 10	16 / 8	2	8

^a^ decayed, missing or filled permanent teeth. ^b^ decayed, missing or filled deciduous teeth

### DNA sequencing information

**Fig. 1:**
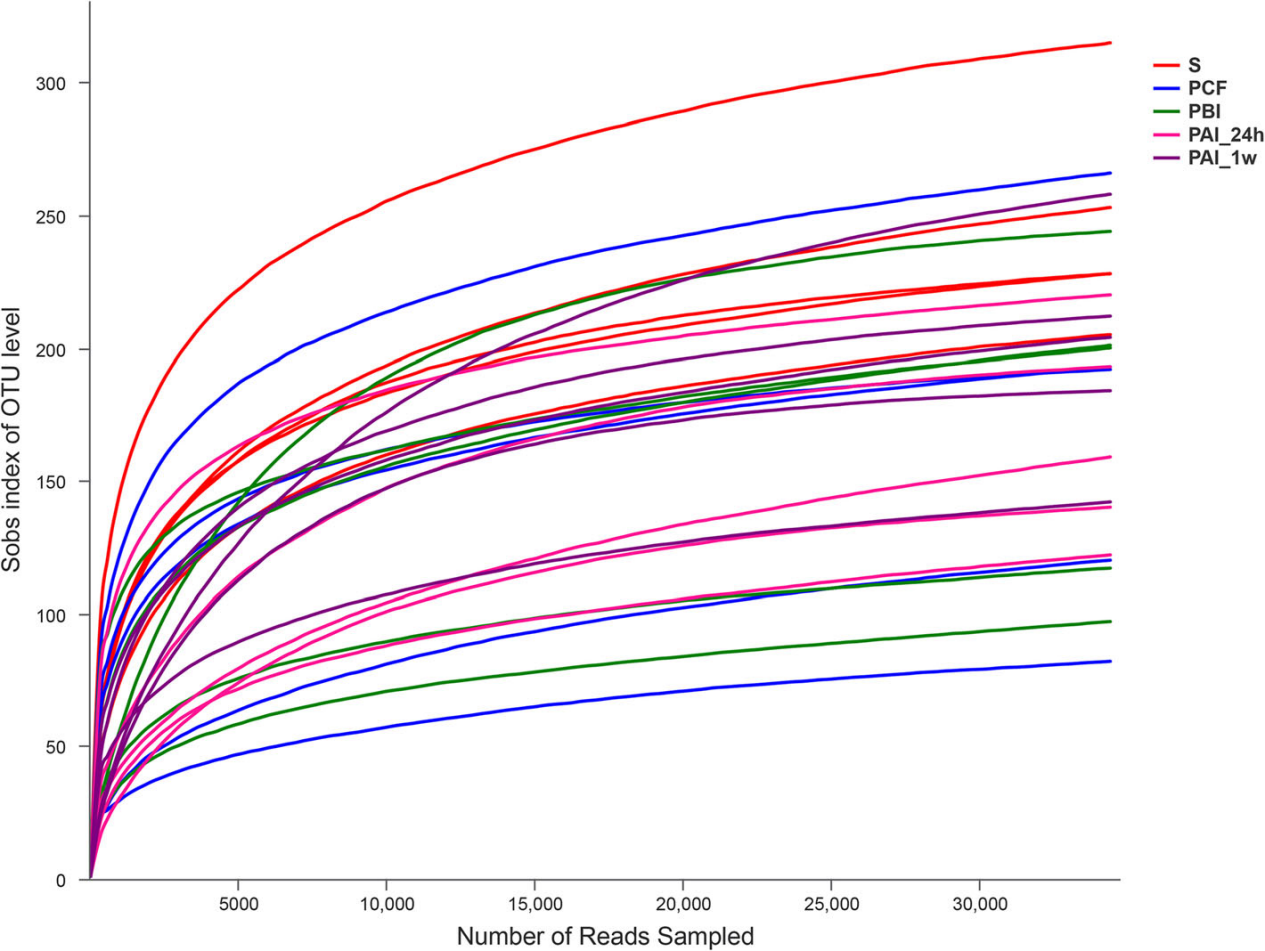
Dilution curves of samples. Each sample is shown separately by a single line. Different color represents different group of samples as indicated in the icons aside. Sobs index refers to the actual observed value of richness, curves were constructed by using the Sobs index of each sample at different sequencing depths

A total of 1,280,788 effective sequences were detected (an average of 51,232 sequences per sample), with an average sequence length of 427 bp. The dilution curve (Fig. 1) tends to be flat, indicating reasonable and representative sequencing data.

### Alpha diversity

**Fig. 2:**
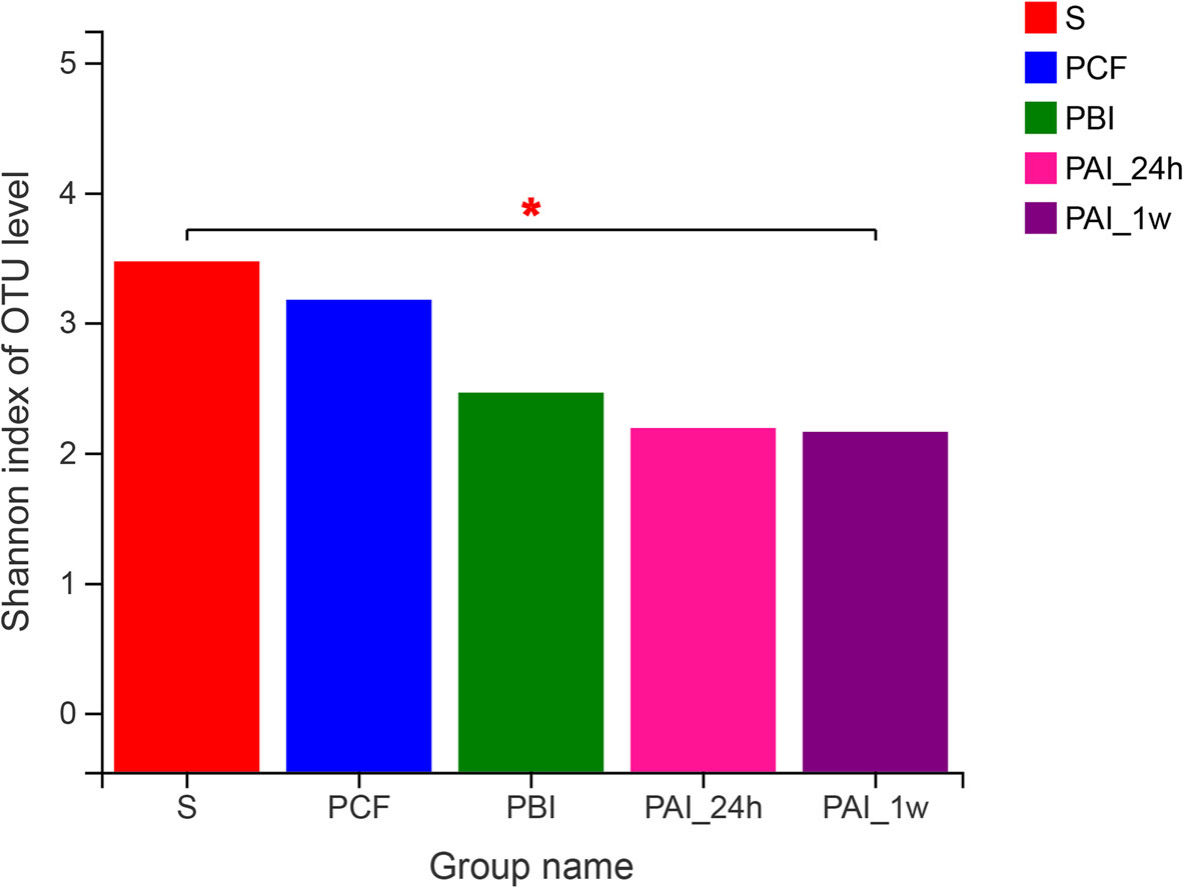
Alpha diversity as measured by the Shannon index. * 0.01 < *P* ≤ 0.05

As shown in Fig. 2, in general, microbial diversity of plaque samples from caries as indicated by Shannon indexes decreased over time after the application of SDF, but no statistically significant difference was found (*P* > 0.05). However, the microbial diversity of PAI-1w were significantly lower than that of saliva samples (*P* < 0.05).

### Microbial community composition and similarity analysis

**Fig. 3:**
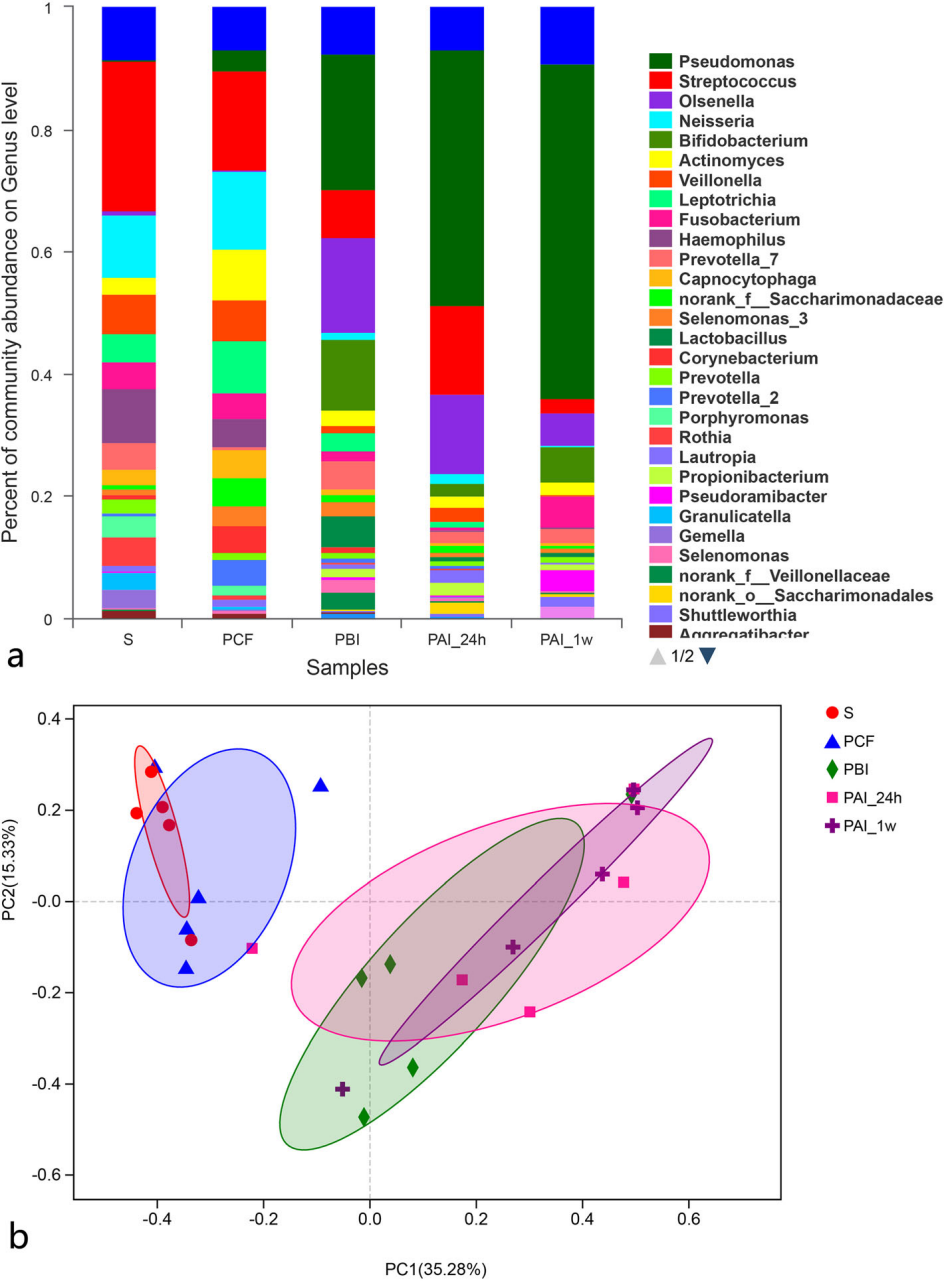
Community bar plot analysis and PCoA diagram. **a** is the Community bar plot, the abscissa is the sample name, and the ordinate is the proportion of species in the sample. **b** is the PCoA diagram on OTU level. The X-axis and Y-axis represent the two selected primary axes, and the percentage represents the explanatory value of the primary axis to the difference in sample composition, the scale of the X-axis and Y-axis is the relative distance, which is meaningless. Dots of different colors or shapes represent samples of different groups. The closer the two sample points are, the more similar is the species composition of the two samples

The microbial community composition of each group of samples was shown in the bar chart (Fig. 3a). The microbial community composition of treated PAI-24 h and PAI-1w samples were noticeably different from that of the untreated PBI group as well as those of saliva and supragingival PCF samples.

Result of PCoA analysis is shown in Fig. 3b, different degrees of overlap (similarity) among the PBI, PAI-24 h and PAI-1w samples, and a small overlap between PCF and saliva samples; however, there was no overlap between the former three and the latter two group of samples. The longer the time after intervention, the smaller the overlap among the samples.

**Fig. 4:**
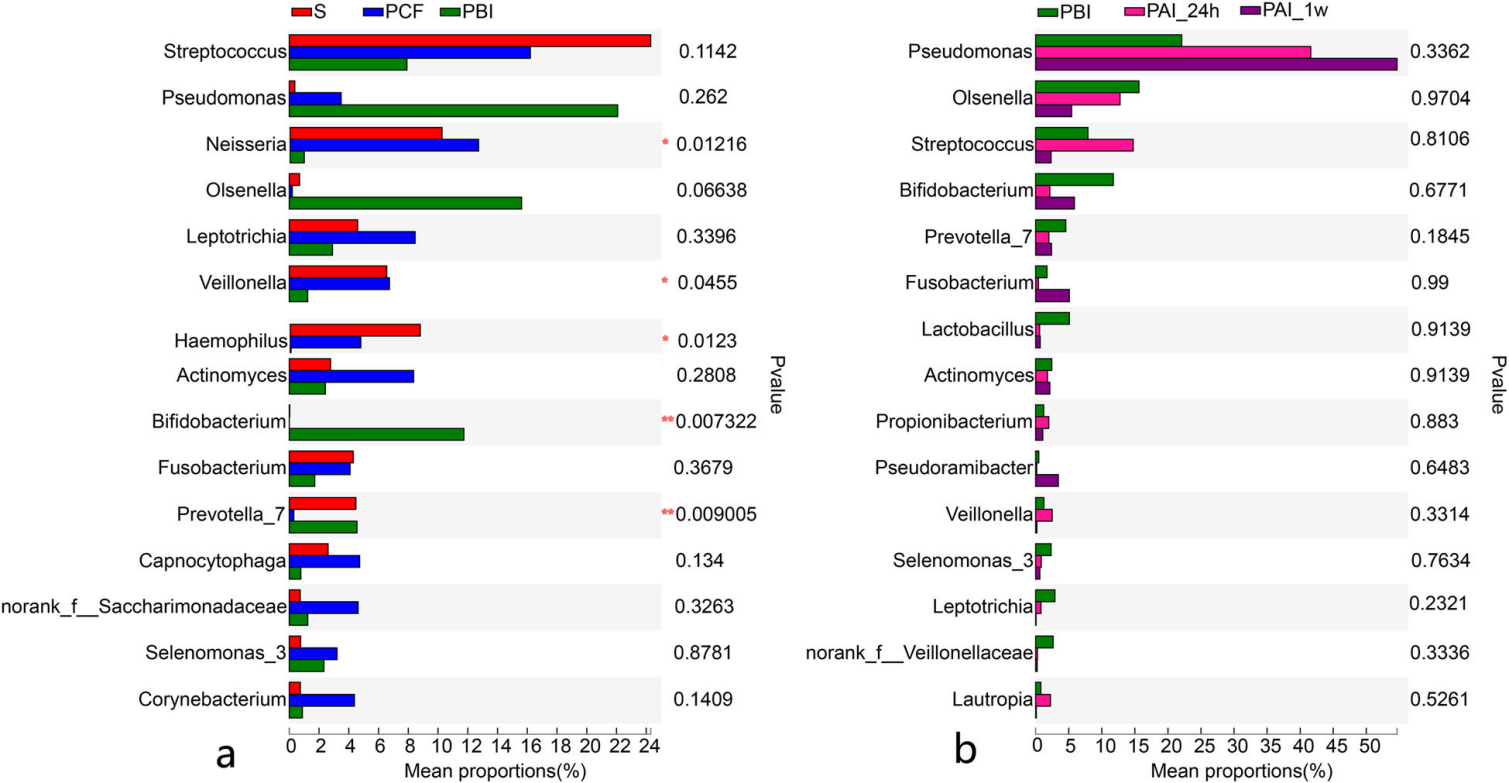
Community composition diversity test on the top 15 richest genera among different groups of samples. **a** is for baseline, **b** is for the application of SDF. * 0.01 < *P* ≤ 0.05, ** 0.001 < *P* ≤ 0.01

The results of the community comparison diversity test (Fig. 4) show that the relative contents of *Pseudomonas*, *Fusobacterium* and *Pseudoramibacter* were noticeably higher in PAI-1w samples compared to PBI samples, indicating they are tolerant to SDF intervention, although not reaching statistical significance(*P* > 0.05). Similarly, other genera, namely *Olsenella*, *Streptococcus*, *Bifidobacterium*, *Prevotella_7*, *Lactobacillus*, *Actinomyces*, *Leptotrichia*, *Selenomonas_3* and *Veillonella* showed a decreasing trend to different degrees, indicating their sensitivity to SDF intervention (*P* > 0.05).

### Network analysis and function prediction

**Fig. 5:**
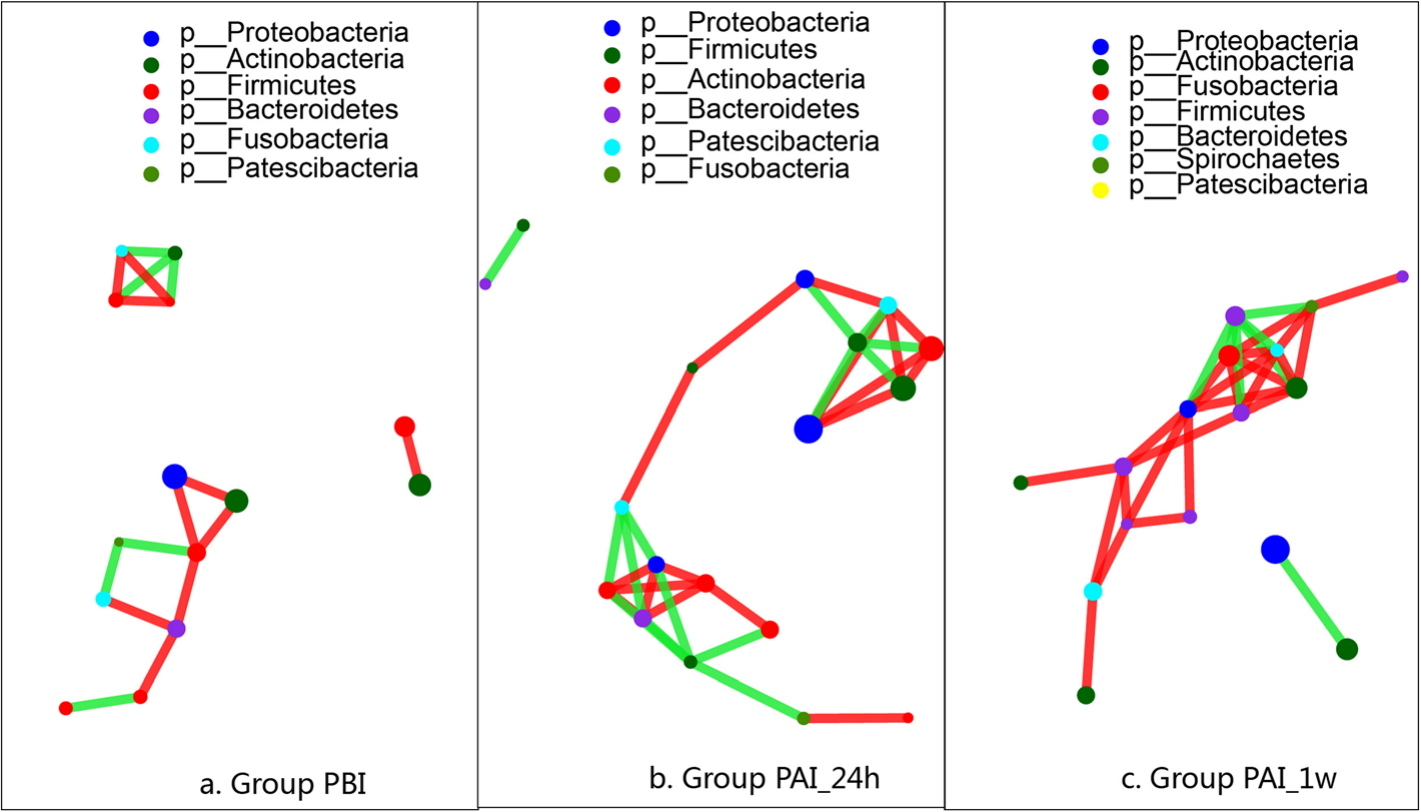
Genus correlation network. The size of the node represents the genus abundance, but different colors of the node represent a different phylum. A line between two nods implies a correlation, red line means positive correlation, green line means negative; the more the lines, the closer the genus is to other genera

Figure 5 is a network diagram made at the genus level, different colors belong to a different phylum. After SDF intervention, the intergeneric connections became more complex and tighter, especially at 1 week after intervention. The positive connections, which indicating collaboration relationships, between genera in the plaque sample after SDF intervention increased compared with that of plaque before intervention, and much more positive associations than negative ones (indicating antagonism relationships) among survived genera was observed at each time point.

**Fig. 6:**
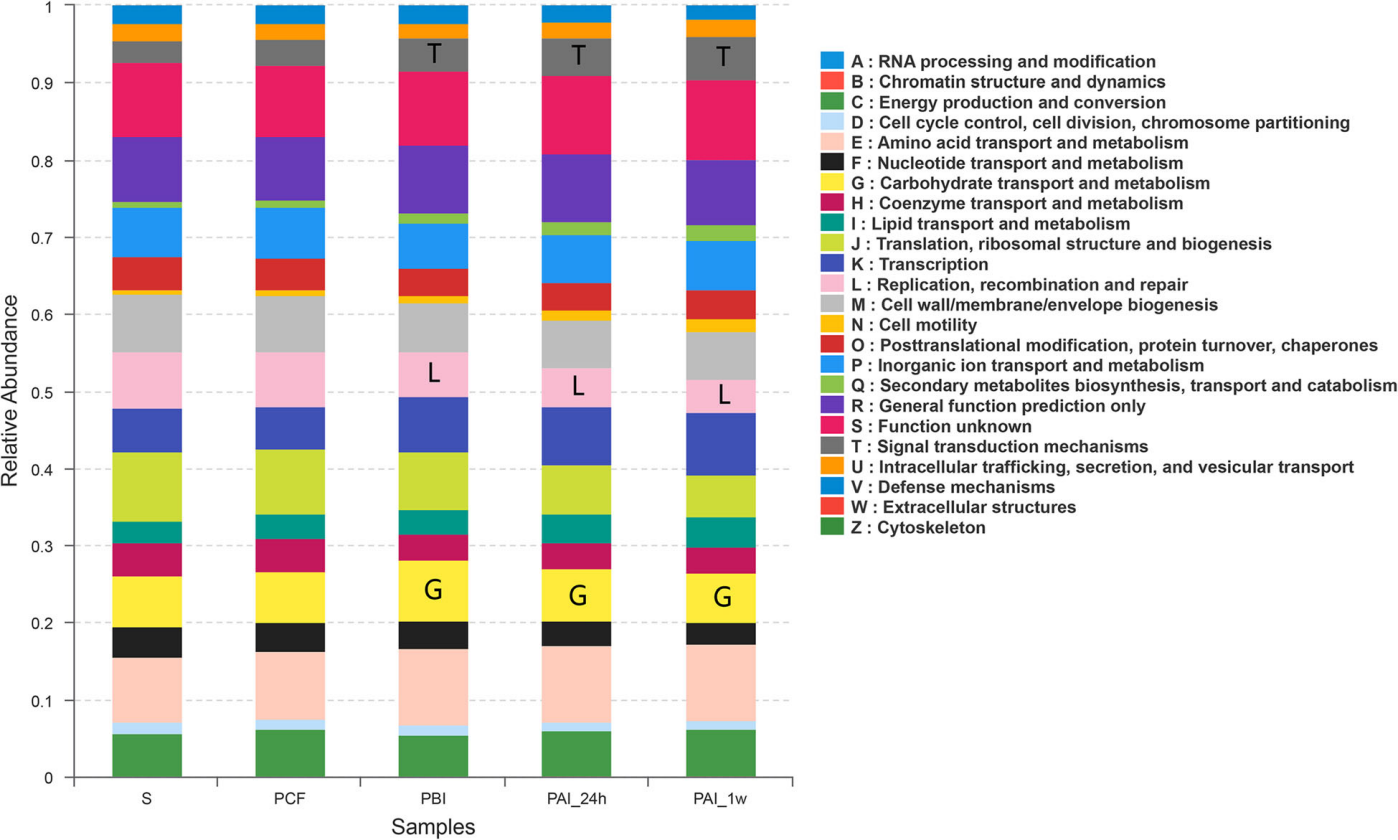
COG function classification

Analysis of the microbial functional characteristics based on the COG (cluster of Orthologous Groups) database was carried out (Fig. 6). It showed that the carbohydrate transportation and metabolic functions (shown in yellow in Fig. 6, G) were significantly (*P* < 0.05) decreased 1 day and 1 week after SDF intervention. The values of relevant functional parameters for PBI, PAI-24 h and PAI-1w samples were 0.080, 0.068 and 0.064, respectively. The value for saliva and PCF samples were 0.067 and 0.066, respectively. The replication as well as recombination and repair functions (shown in pink in Fig. 6, L) were also significantly reduced, and the signal transduction mechanism (shown in gray in Fig. 6, T) was significantly increased.

## Discussion

The antimicrobial effect of SDF has been proven by various previous studies [15–20, 25], but the effect of SDF on micro-ecology of dental plaque has seldom been explored. Based on the already known antimicrobial effect of SDF, this pioneer preliminary study aimed to further our knowledge of SDF on dental plaque from the micro-ecosystem aspect.

In this study, extracted deciduous teeth with large caries from school-age children were collected and topically treated in vitro with a 38% SDF solution. Dental plaque from the caries pre- and post-intervention were collected for microbial sequencing and the results were compared. The amount of 9 out of 15 richest bacteria genera, the microbial diversity and the microbial community composition in the dental plaque after SDF intervention were noticeably changed. The relative proportions of *Pseudomonas*, *Fusobacterium* and *Pseudoramibacter* were higher than before the intervention, although no significant different was found (*P* > 0.05). After SDF intervention, microbial association in the dental plaque became more complex with positive connections overwhelming the negative ones. Carbohydrate transportation and metabolic functions in the dental plaque were significantly reduced (*P* < 0.05).

The microbial diversity was reduced, the microbial richness as well as microbial community composition was shifted post 38% SDF intervention, which supported the broad spectrum bactericidal effect as shown in previous studies [25], although no statistically significant difference was found in this study. A recent study on 38% SDF, which compared pre- and one-month-post-intervention dental plaque sample from the mouth of adult patients [29], found no significant difference in the plaque microorganisms composition either. However, difference in dentition, sampling conditions and the follow-up interval should also be noted. The statistical indifference in this study may also be due to the relatively small sample size adopted in this study. Based on experiences of previous studies, a large difference in the microbial diversity before and after SDF intervention was estimated, yielding a number of five children needed in this study. And this relatively small sample size was also practical if balancing the research expenses. Clearly, a larger sample with more funding should be adopted in future studies.

Most of the genera present before the intervention (*Olsenella*, *Streptococcus*, *Bifidobacterium*, *Prevotella_7*, *Lactobacillus*, *Actinomyces*, *Leptotrichia*, *Selenomonas_3*, *Veillonella*) were sensitive to SDF, and the relative amounts decreased substantially. This was consistent with previous studies [15, 16, 18, 20] and supported the broad spectrum bactericidal effect of SDF [25]. *Streptococcus*, *Lactobacillus* and *Actinomyces* have long been recognized as classical cariogenic bacteria. *Prevotella_7*, *Selenomonas_3*, and *Bifidobacterium* are often found in deep dentin caries [30]. *Olsenella*, which could produce lactic acid, seldom mentioned in the past, have recently been found associated with dentin and root caries [31, 32]. *Leptotrichia* has a strong glycolysis effect [33], and its detection rate is increased in the process of caries [34, 35]. *Veillonella* has a unique intracellular pH control mechanism [36], which might promote the cariogenicity of *Streptococcus mutans* [35, 37]. By inhibiting these bacteria, cariogenicity of the dental plaque may be suppressed, creating a new microbial balance.

It was also found that *Pseudomonas*, *Fusobacterium* and *Pseudoramibacter* could withstand the effect of topically applied SDF. The possible role of *Pseudomonas* in dental caries was indicated recently. NavNeet Kaur et al. [38] detected a high level of *Pseudomonas* in the dentin caries. In Liang’s study [39], *Pseudomonas* was mentioned as a potential pathogen of caries, but suggested the relationship be further studied. *Pseudomonas* is a non-fermenting, obligate aerobic Gram-negative bacillus. It has no special nutritional requirements and is common in soil, fresh water, seawater. A total of 29 species have been identified, of which at least three are pathogenic to animals or humans, causing infections. *Pseudomonas aeruginosa* is part of the commensal oral flora, but can become a pathogen and cause nosocomial infection [40]. Small infective doses of the bacteria can produce local abscesses; large doses can lead to death from systemic infection. *Fluorescent pseudomonas ssp.* can cause spoilage of frozen meat, eggs, milk and dairy products. *Pseudomonas birensis* can be transmitted through the mouth, the respiratory tract or in wounds. *Pseudoramibacter* is a nonmotile, nonsporeforming, strictly anaerobic, Gram-positive bacillus, which was saccharolytic. The end products of its fermentation are formate, acetate, butyrate, caproate, and hydrogen [41]. It can cause periapical infection [42–44], it often found in deep dentin caries [30, 45] and infected root canals [41]. Similarly, *Fusobacterium* is often detected in deep dentin caries [30, 45] and infected root canals [43, 44, 46]. It is a strictly anaerobic Gram-negative bacillus living in the oral cavity and the upper digestive, intestine and urogenital tracts of humans or animals, as well as in the soil. It is most commonly seen in oral dental plaque. Most of the strains do not ferment any sugars, only a few strains are known to weakly ferment glucose and fructose. Briefly, *Pseudomonas*, *Fusobacterium* and *Pseudoramibacter* may not play important role in the development of dental caries, but they are more closely related to root canal or periapical infections. In this study, deciduous teeth with extensive caries or obvious dental pulp symptoms from school-age children was studied, which could explain the richness of these bacteria in the sample. The results would also provide guiding information in the prevention and control of extensive deciduous caries associated with pulp infection or periapical disease. The tolerance of these three kinds of bacteria to single time SDF application in this in vitro study should be pay cautious attention to when use SDF in the clinical situation in child population. Further studies are worth conducting and should be designed according to this special concern in order to form a clear guide.

The functional characteristics of plaque in caries were also compared and analyzed based on the COG database for homologous classification of gene products. Carbohydrate transportation and metabolism function in plaque were significantly reduced 24 h and 1 week after intervention (shown in yellow in Fig. 6, G), and the replication, recombination and repair function in plaque (shown in pink in Fig. 6, L) were also significantly reduced. These changes revealed from another aspect the overall inhibitory effect of SDF on the bacterial community in dental plaque, which affected the properties of the whole plaque, especially the cariogenicity within the plaque micro-ecology. The function of signal transduction mechanism was significantly increased, which could be correlated with the enhancement of synergistic effect between residual bacteria in plaque after intervention.

There are limitations in this study. First of all, this preliminary study focused on investigating micro-ecological changes in dental plaque on extensive caries of deciduous teeth after topical SDF treatment, mainly before-after comparison was adopted. However, if untreated placebo control as well as well-defined positive control groups were there to make comparisons between groups, it would have been ideal to monitor possible affecting and confounding factors to the observed change effects well. Secondly, restricted by ethic issues, extracted deciduous teeth were used in this study. As a result, intervention and sampling were all carried out in vitro. An in vitro study differs from the real clinical situation. The effect of common oral flora, saliva, food and environmental microorganisms on the formation of new microbial balance could not be reproduced or evaluated. New micro-ecological balance formation after SDF intervention in this study could only be based on the before-intervention status as well as the effect of SDF. Bacteria which were sensitive to SDF were controlled, while those with relatively high tolerance to SDF grew relatively better after the intervention. After SDF intervention, the microbial association in the dental plaque became more complex with positive connections overwhelming the negative ones, indicating that the connections and collaborations among the remaining bacterial communities were enhanced. It also had to be considered whether these changes may also be related to the medium and growth conditions (such as ambient air rather than 5% CO2 or anaerobic). An untreated control would have been ideal to help monitor changes in the microbes based on the culture conditions. Thirdly, the observation period in this study was relatively short which may have its limitations. Most parameters in this study showed that the greatest microbial difference was found at 1 week after SDF intervention, indicating a possible time effect of SDF, longer study period should be suggested for future investigations. In summary, further a longer follow-up period study with sophisticated control designs which can mimic the real mouth environment or a real mouth situation should be performed to help deepen the knowledge of the dental plaque micro-system.

## Conclusions

This in vitro study focused on the effect of 38% SDF on the dental plaque micro-system within carious lesions over time. Topical application of SDF had an inhibitory effect on cariogenic organisms which led to reduced carbohydrate metabolism within the surviving bacteria. The microbial diversity decreased with increased positive correlations among surviving bacteria demonstrating a redistribution of the plaque micro-ecology. These findings provide an increased understanding of the caries prevention and control mechanism of SDF at the dental plaque micro-system level.

## Data Availability

The datasets used and/or analysed during the current study are available from the corresponding author on reasonable request.

## Abbreviation

SDF: Silver diammine fluoride
